# Retraction: Exogenous salicylic acid-induced drought stress tolerance in wheat (*Triticum aestivum* L.) grown under hydroponic culture

**DOI:** 10.1371/journal.pone.0284669

**Published:** 2023-04-19

**Authors:** The PLOS ONE Editors

The *PLOS ONE* Editors retract this article [1, 2] because it was identified as one of a series of submissions for which we have concerns about authorship, competing interests, and peer review. We regret that the issues were not addressed prior to the article’s publication.

AA, ZA, AR, HMA, MHS, MZMS, and CH did not agree with the retraction. MN, SH, TJ, SA, RS, SA, TS, and MAJ either did not respond directly or could not be reached.
